# Effort-Reward Imbalance in Emergency Department Physicians: Prevalence and Associated Factors

**DOI:** 10.3389/fpubh.2022.793619

**Published:** 2022-02-07

**Authors:** Mengge Tian, Xuan Zhou, Xiaoxv Yin, Nan Jiang, Yafei Wu, Jiali Zhang, Chuanzhu Lv, Yanhong Gong

**Affiliations:** ^1^Department of Social Medicine and Health Management, School of Public Health, Tongji Medical College, Huazhong University of Science and Technology, Wuhan, China; ^2^Department of Anesthesiology, Tongji Hospital, Tongji Medical College, Huazhong University of Science and Technology, Wuhan, China; ^3^Department of Emergency Medicine, Sichuan Provincial People's Hospital, University of Electronic Science and Technology of China, Chengdu, China; ^4^Research Unit of Island Emergency Medicine, Chinese Academy of Medical Sciences, Hainan Medical University, Haikou, China; ^5^Key Laboratory of Emergency and Trauma of Ministry of Education, Hainan Medical University, Haikou, China

**Keywords:** emergency department, physicians, effort-reward imbalance, workplace violence, mental health

## Abstract

**Objectives:**

To examine the prevalence of effort-reward imbalance and explore its associated factors among emergency department physicians in China.

**Methods:**

A cross-sectional survey was conducted in the Chinese emergency department in 2018. A total of 10,457 emergency department physicians completed a structured questionnaire containing demographic characteristics, work-related data, and effort-reward imbalance scale. All the data were analyzed using descriptive analysis and stepwise logistic regression.

**Results:**

The prevalence of effort-reward imbalance was 78.39% among emergency department physicians in China. The results showed that the male emergency department physicians with a bachelor's degree, an intermediate title, long years of service, a high frequency of night shift, and who suffered workplace violence were at a higher risk of effort-reward imbalance. In contrast, physicians with higher monthly income and perceived adequate staff were associated with a lower risk of effort-reward imbalance.

**Conclusions:**

The situation of effort-reward imbalance was serious among emergency department physicians in China. Administrators should pay more attention to key groups and take measures from the perspectives of effort and reward to improve the effort-reward imbalance in emergency department physicians.

## Introduction

Emergency department (ED) physicians are crucial in the emergency medical service system. However, due to the special working environment of ED, ED physicians are under high time pressure, workload, and psychological pressure (1). On the one hand, this is since ED mainly treats patients with acute and critical illness, and on the other hand, it is because ED is an open and complicated department; it is more chaotic and prone to violence incidences (1–3). A previous study revealed that ED physicians faced a higher level of work stress than general ward physicians (4).

Effort-reward imbalance (ERI) is a classical theory of work stress, which asserts that the imbalance between effort and reward of work (e.g., salary, promotion prospects, job security, and self-esteem) will evoke great negative emotions in employees, thereby causing intense stress reaction (5). Nowadays, the theory of ERI has been widely applied to the physicians' group. Previous studies in Japan (6) and Switzerland (7) reported that the prevalence of ERI in physicians was 57% and 64.9%, respectively. However, studies in Germany (8) and Norway (9) on physicians showed that the proportion was 25.1% and 10.3%, respectively. The prevalence of ERI in physicians varied widely between countries and regions, which may be relevant to differences in health care systems and cultural backgrounds. Because of the particularities of ED, ED physicians may face a more severe situation of ERI. However, up to now, the occurrence of ERI in ED physicians has been seldom studied. As an adverse working condition, the imbalance between high effort and low reward has a series of negative effects on employees' health and work performance (7, 10–13). Studies have shown that ERI was highly associated with chronic fatigue (11), cardiovascular disease (10), and depression (12). Besides, it was also related to increased turnover intention (7) and sickness absence (13). Therefore, it is necessary to explore the current situation and related factors of ERI in ED physicians.

In China, emergency medical services cover various fields, including pre-hospital care, disaster medicine, and in-hospital care. And these services play an important role in major medical treatment and multidisciplinary diagnosis and treatment (14). At present, Chinese hospitals have established EDs, and China has formed a relatively complete emergency medical system (14). However, with the rapid increase in emergency service demands, overcrowding in ED has become increasingly severe in China (15). According to the research, Chinese emergency patients exceeded 166.5 million in 2017 (16). EDs still face the problem of insufficient numbers of physicians and heavy workloads (17). The above reasons may make Chinese ED physicians more prone to ERI. Thus, this large cross-sectional study was conducted among ED physicians in China, aiming to understand the prevalence of ERI and explore its associated factors in Chinese ED physicians, which would provide an empirical basis for the improvement of ERI among ED physicians.

## Methods

### Ethics Statement

This study was designated as exempt human research by the Research Ethics Committee in Hainan Medical University (HYLL-2018-035). Each physician was voluntary, and all the data would keep confidential.

### Study Design

This was a part of a national, cross-sectional study on emergency medical resources in China. We conducted this online survey from July to August 2018 in coordination with the Medical Administration Bureau of the National Health Commission of the People's Republic of China. The questionnaire link was distributed to the ED physician work platform of the pre-hospital emergency facility configuration monitoring department, inviting ED physicians to participate in the survey. The questionnaire link was posted to the work platform every seven days to alert ED physicians to participate in the survey until the investigation was complete. Participants took part in the survey through access to the link, but all participants were required to read and agree to the electronic informed consent statement before answering. The data were administered by an online survey platform in China (platform name: Questionnaire Star, website: https://www.wjx.cn).

In this study, we included physicians who worked in ED and volunteered to participate in the survey and excluded those interns who had not yet obtained a practicing certificate. In all, 15,288 physicians clicked the questionnaire link, and 10,457 physicians completed the questionnaire. The response rate was 68.4%.

### Measurements

Data were collected using a standard structured questionnaire developed based on literature review and pre-tested in a pilot study of 30 ED physicians to ensure that the questions were clear and understandable (5, 6, 8, 9). The questionnaire consisted of three parts in this study: sociodemographic characteristics, work-related factors, and ERI.

The sociodemographic characteristics covered sex, age, marital status, and educational level. Work-related factors included professional title, monthly income, years of service, frequency of night shift, perceived shortage of physicians, frequency of suffering from workplace verbal and physical violence in the past year. Among these factors, educational level was categorized as “associate degree or vocational diploma,” “bachelor degree,” “master degree or above,” In China, an associate degree or vocational diploma indicates 12–15 years of education. A bachelor's degree indicates 17 years of education. A master's degree or above refers to 20 years and above of education. The perceived shortage of physicians was examined by one question: “Do you think the current number of physicians in ED can meet the needs of daily work?” (“Not meet demands,” “General,“ “Meet demands”).

In the third part of the questionnaire, ERI was assessed by the Chinese version of the ERI scale (18, 19), which originated from Siegrist in 1996 (5). The Chinese version of the ERI questionnaire has good reliability and validity in Chinese medical staff, and the Cronbach's alpha of the effort and reward subscales is 0.78 and 0.81, respectively (19). We used two subscales of the ERI to evaluate the balance between job effort and job reward. Job effort included six items (time pressure, interrupting work, responsibility, extra work, physical labor, and increased workload), with a total range from 6 to 30. Every item was scored on a 5-point Likert scale ranging from 1 (strongly disagree) to 5 (strongly agree). Job reward contained 11 items, including three dimensions of esteem, job promotion, and job security. In this subscale, every item was rated on a 5-point Likert scale ranging from 1 (strongly agree) to 5 (strongly disagree), and the total score was between 11 to 55. Based on the above, the effort-reward ratio (ERR) was calculated according to the formula: ERR = (11^*^effort)/(6^*^reward) (20). When the value of ERR is beyond 1.0, it indicates that ED physicians experienced the ERI (20). In this study, job effort had a Cronbach's alpha value of 0.86, and job reward had a Cronbach's alpha value of 0.93.

### Statistical Analysis

All data were analyzed using the Statistical Analysis System (SAS) version 9.4 for Windows (SAS Institute Inc., Cary, NC, USA). Firstly, Chi-square tests were conducted to describe sociodemographic characteristics and work-related factors in different effort-reward status groups. All the categorical variables were reported using frequencies and percentages. As appropriate, continuous variables were compared using the Student's *t*-test or Mann-Whitney *U* test. The continuous variables were reported using mean (standard deviation) or median (25th−75th percentiles). Then, a stepwise logistic regression was conducted to assess the association between variables and the two groups of effort-reward status. The results were reported by adjusted odds ratios (ORs) and 95% confidence intervals (95% CIs) for each variable. We used Spearman's Rho correlations to examine multicollinearity between independent variables. Spearman's Rho correlations were <0.7, indicating that no multicollinearity was detected (Supplementary Table 1). The statistical significance was at a *P*-value of < 0.05.

## Results

### Characteristics of the Participants

The general characteristics of ED physicians are presented in Table 1. Among 10,457 ED physicians, the median age was 31, 72.98% were male, 27.02% were female. Of the participants, most had a bachelor's degree, accounting for 74.49%. The marital status of ED physicians was mainly married, accounting for 84.42%. Only 13.13% of ED physicians had senior titles. About half of them had more than five years of service, and 53.87% had 6–10 times night shifts per month. More than two-thirds of ED physicians (73.32%) perceived that the number of physicians was inadequate in ED. About workplace violence, 81.81% of ED physicians suffered verbal violence, and 27.63% suffered physical violence in the past year.

**Table 1:** Comparisons of characteristics in different effort-reward status groups among physicians in the emergency department.

	**Total**	**Effort-reward balance**	**Effort-reward imbalance**	**χ^2^**	***P***
	***N (%)***	***N (%)***	***N (%)***		
Total	10,457 (100)	2,260 (21.61)	8,197 (78.39)		
Sociodemographic characteristics					
Age^&^	31 (28–35)	34 (29–40)	36 (31–42)	*Z* = −9.20	<0.001^**^
Sex				146.81	<0.001^**^
Male	7,632 (72.98)	1,423 (62.96)	6,209 (75.75)		
Female	2,825 (27.02)	837 (37.04)	1,988 (24.25)		
Educational level				47.54	<0.001^**^
Associate degree or vocational diploma^#^	1,684 (16.10)	447 (19.78)	1,237 (15.09)		
Bachelor degree	7,789 (74.49)	1,557 (68.89)	6,232 (76.03)		
Master degree or above	984 (9.41)	256 (11.33)	728 (8.88)		
Marital status				50.00	<0.001^**^
Unmarried/other	1,629 (15.58)	1,800 (79.65)	7,028 (85.74)		
Married	8,828 (84.42)	460 (20.35)	1,169 (14.26)		
Work-related factors					
Title				126.92	<0.001^**^
Junior or less	4,972 (47.55)	1,307 (57.83)	3,665 (44.71)		
Intermediate	4,112 (39.32)	686 (30.35)	3,426 (41.80)		
Senior	1,373 (13.13)	267 (2260)	1,106 (13.49)		
Monthly income (YUAN)				8.72	0.013^*^
≤4,000	3,862 (36.93)	850 (37.61)	3,012 (36.75)		
4,001~6,000	3,562 (34.06)	714 (31.59)	2,848 (34.74)		
≥6,001	3,033 (29.00)	696 (30.80)	2,337 (28.51)		
Years of service				213.29	<0.001^**^
<1	1,448 (13.85)	484 (21.42)	964 (11.76)		
1–5	3,965 (37.92)	949 (41.99)	3,016 (36.79)		
6–10	2,458 (23.51)	412 (18.23)	2,046 (24.96)		
≥11	2,586 (24.73)	415 (18.36)	2,171 (26.49)		
Frequency of night shift (per month)				178.39	<0.001^**^
0~5	2,033 (19.44)	641 (28.36)	1,392 (16.98)		
6~10	5,633 (53.87)	1,183 (52.35)	4,450 (54.29)		
≥11	2,791 (26.69)	436 (19.29)	2,355 (28.73)		
Perceived shortage of physicians				852.27	<0.001^**^
Not meet demands	7,667 (73.32)	1,132 (50.09)	6,535 (79.72)		
General	1,856 (17.75)	673 (29.78)	1,183 (14.43)		
Meet demands	934 (8.93)	455 (20.13)	479 (5.84)		
Workplace verbal violence (times)				892.34	<0.001^**^
0	1,902 (18.19)	835 (36.95)	1,067 (13.02)		
1~3	4,130 (39.50)	964 (42.65)	3,166 (38.62)		
≥4	4,425 (42.32)	461 (20.40)	3,964 (48.36)		
Workplace physical violence				310.01	<0.001^**^
Yes	2,889 (27.63)	293 (12.96)	2,596 (31.67)		
No	7,568 (72.37)	1,967 (87.04)	5,601 (68.33)		

* *P-value < 0.05*,

** *P-value < 0.001*.

& *Considering the non-normal distribution of age, it was compared by Mann-Whitney U test and reported using median (25th−75th percentiles)*.

# *An associate degree refers to students graduating from senior middle school (grade year 10 to year 12) receiving 3 years of education in college, or students graduating from junior middle school (grade year 7 to year 9) receiving 5 years of education in college*.

*A vocational diploma refers to students graduating from senior middle school receiving 2 years of education in vocational schools or students graduating from junior middle school receiving 3 years of education in vocational schools*.

### The Prevalence of ERI and Its Related Factors

Of the respondents, 8,197 (78.39%) experienced ERI. The relationships between demographic characteristics or work-related factors and ERI are shown in Table 1. There were statistically significant differences in the prevalence of ERI among all the groups of demographic characteristics and work-related factors (*P* < 0.05). To present the data more comprehensively, we also conducted the univariate analysis of the effort-reward ratio values, which was shown in Supplementary Table 2.

The independent factors associated with ERI are shown in Table 2. The ERI was significantly associated with being male (*OR* = 1.45, 95%CI: 1.30–1.62). ED physicians with bachelor's degrees were more likely to experience ERI than those with associate degrees or vocational diplomas (*OR* = 1.20, 95%CI: 1.05–1.38). In contrast, ED physicians with intermediate titles had higher risks of ERI than those with junior or lower titles (*OR* = 1.45, 95%CI: 1.28–1.64). In the aspect of monthly income, ED physicians with a monthly income of 6001 yuan or more would be less likely to experience ERI (*OR* = 0.68, 95%CI: 0.60–0.78). On the contrary, ED physicians who had long years of service (*OR* = 1.57, 95%CI: 1.30–1.90), more than 11 times night shifts every month (*OR* = 1.41, 95%CI: 1.21–1.65), had higher risks of exhibiting ERI. Compared to ED physicians with a perceived staff shortage, when the staffs were adequate, the risk of ERI in ED physicians markedly decreased (*OR* = 0.28, 95%CI: 0.24–0.33). In addition, in terms of workplace violence, whether verbal violence (*OR* = 3.61, 95%CI: 3.11–4.21) or physical violence (*OR* = 1.57, 95%CI: 1.35–1.82) could significantly increase the risk of ERI among ED physicians.

**Table 2:** Binary logistic regression examining factors associated with ERI.

	**OR (95%CI)**	***P***
Sex		
Female	1.00 (reference)	
Male	1.45 (1.30–1.62)	<0.001^**^
Educational level		
Associate degree or vocational diploma^#^	1.00 (reference)	
Bachelor degree	1.20 (1.05–1.38)	0.010^*^
Master degree or above	1.15 (0.93–1.41)	0.189
Title		
Junior or less	1.00 (reference)	
Intermediate	1.45 (1.28–1.64)	<0.001^**^
Senior	1.20 (0.99–1.45)	0.063
Monthly income (YUAN)		
≤4000	1.00 (reference)	
4,001~6,000	0.91 (0.80–1.03)	0.122
≥6,001	0.68 (0.60–0.78)	<0.001^**^
Years of service		
<1	1.00 (reference)	
1–5	1.11 (0.96–1.29)	0.155
6–10	1.42 (1.19–1.69)	<0.001^**^
≥11	1.57 (1.30–1.90)	<0.001^**^
Frequency of night shift (per month)		
0~5	1.00 (reference)	
6~10	1.11 (0.98–1.27)	0.100
≥11	1.41 (1.21–1.65)	<0.001^**^
Perceived shortage of physicians		
Not meet demands	1.00 (reference)	
General	0.40 (0.36–0.45)	<0.001^**^
Meet demands	0.28 (0.24–0.33)	<0.001^**^
Workplace verbal violence (times)		
0	1.00 (reference)	
1~3	1.93 (1.70–2.19)	<0.001^**^
≥4	3.61 (3.11–4.21)	<0.001^**^
Workplace physical violence		
Yes	1.57 (1.35–1.82)	<0.001^**^
No	1.00 (reference)	

* *P-value < 0.05*,

** *P-value < 0.001*.

# *An associate degree refers to students graduating from senior middle school (grade year 10 to year 12) receiving 3 years of education in college, or students graduating from junior middle school (grade year 7 to year 9) receiving 5 years of education in college*.

*A vocational diploma refers to students graduating from senior middle school receiving 2 years of education in vocational schools or students graduating from junior middle school receiving 3 years of education in vocational schools*.

## Discussion

This study described the current situation of ERI and explored its associations with demographic characteristics and work-related factors among ED physicians in China. Our study found that 78.39% of Chinese ED physicians experienced ERI. The proportion was higher than the prevalence of 21.9%–64.9% in general physicians (6–8, 21), which reflected that ED physicians bore a higher level of work stress than general physicians. However, due to the lack of international investigation of ERI among ED physicians, it was hard to make comparisons among different countries. A multinational study on the prevalence of ERI among ED physicians needs to be carried out to understand its occurrence and severity worldwide.

Previous studies have shown that gender differences existed in the prevalence of ERI among medical workers, but the conclusions have not been unified (22). In our study, male ED physicians had a higher prevalence of ERI than females. A survey of Chinese medical workers in the in-patient wards also found that ERR in male medical workers was higher (19). However, studies among physicians in Greece (23) and Sweden (24) pointed out no gender difference in ERR, inconsistent with our result. The difference may be related to different social backgrounds and cultures in various countries. In China, traditional gender role attitudes and motivations expose males more often to work stress of success and react by high effort, which increases the risk of ERI among male ED physicians (19).

In addition, we found that ED physicians with bachelor's degrees were more likely to experience ERI, which might be determined by the context of the medical professions. The medical industry had high demands for knowledge and ability, and the competition was also intense (25). In our study, ED physicians with bachelor's degrees were in the middle level. Compared to ED physicians with low educational levels, ED physicians with bachelor's degrees had to do more work to achieve their own higher job expectations. Still, their rewards were more limited than ED physicians with high educational levels (26). This study also found that ED physicians with intermediate titles were more prone to ERI. Yan et al. obtained similar results that ED physicians with intermediate titles were more likely to suffer from burnout (27). In Chinese hospitals, physicians with intermediate titles are the backbone of medical services. They not only have to engage in busy clinical diagnosis and treatment work but also undertake heavy teaching and scientific research tasks (28, 29). Moreover, the proportion of physicians with intermediate titles is higher than that with senior titles, and the number of physicians with intermediate titles is relatively large (30). Therefore, physicians with intermediate titles must work harder to get promoted (30). These intertwined pressures make them more prone to ERI. This suggested that hospital administrators should pay more attention to ED physicians with bachelor's degrees or intermediate titles, take measures such as reducing workload, and providing opportunities for further training to improve the situation of ERI.

Work-related factors were also considered as the important associated factors of ERI. According to our study, ED physicians with long years of service had a higher risk of ERI. One possible explanation may be that ED physicians of seniority have endured long work hours and frequent call duty, which could result in greater emotional exhaustion and fatigue, thereby leading to a higher sense of job efforts (31). Higher frequencies of night shifts could also increase the risk of ERI. Previous studies on the night shift confirmed that in addition to increasing work stress of ED physicians, frequent night shift could also lead to sleep disorders and have hazards on their physical and mental health, which was unfavorable to the balance of effort and reward for ED physicians (32, 33). Besides, our study also illustrated that perceived adequate staffs and monthly income of 6001 yuan or more were protective factors for ERI of ED physicians. Sufficient staff in ED could reduce the workload of ED physicians (34). Higher monthly income could allow ED physicians to perceive more rewards. Both above two factors helped reduce the risk of ERI for ED physicians. In consequence, hospital administrators should focus on ED physicians with long years of service, schedule night shift scientifically, replenish ED physicians timely, and establish a proper salary system to reduce the level of ERI in ED physicians.

Workplace violence was also a risk factor for ERI in ED physicians. ED was at a high risk of workplace violence (35). Both 81.81% incidence of verbal violence in this study and 78.1% incidence of workplace violence in another study among Turkish ED physicians confirmed the high incidence of workplace violence in ED (36). A previous study has also found that verbal violence or physical violence significantly increased the risk of ERI among ED physicians (37). This suggests that hospital administrators should strengthen hospital security work to reduce the frequency of workplace violence. At the same time, timely psychological counseling should be conducted for ED physicians who encounter workplace violence to reduce the adverse impact of workplace violence on the ERI of ED physicians.

### Strengths and Limitations

To our knowledge, this is the first national cross-sectional study on ERI among ED physicians in China. But the study also has some limitations. Firstly, the cross-sectional design of this study could not provide definitive causal conclusions, and further prospective studies are needed. Secondly, the participants in this study were only ED physicians, who were not representative of all physicians in China; therefore, the results of our study should be generalized with caution. Finally, more related factors of ERI among ED physicians, such as family-related factors, need to be explored.

## Conclusion

ERI was highly prevalent among ED physicians in China, especially males, who had bachelor's degrees, intermediate titles, and long years of service. While paying attention to these focus groups, hospital administrators should reduce the efforts of ED physicians by increasing the number of ED physicians, optimizing night shift schedules, and tightening security. At the same time, ED physicians should be strongly supported in skills training, salary, and promotion, to reduce the imbalance between effort and reward for ED physicians.

## Data Availability Statement

The datasets used and/or analyzed during the current study are available from the corresponding author on reasonable request.

## Ethics Statement

The studies involving human participants were reviewed and approved by the Research Ethics Committee in Hainan Medical University, Haikou, China (HYLL-2018-035). All participants provided informed consent. Moreover, each physician was voluntary, and all the data would keep confidential.

## Author Contributions

MT, CL, and YG designed this study. CL and XY collected the data. XZ, NJ, YW, and JZ completed data analysis. CL, XZ, and YG supervised all the processes in this study. MT and XZ drafted the main manuscript text. All authors reviewed and approved the manuscript.

## Funding

This study was supported by the Department of Science and Technology of Hainan Province, Major Science and Technology Projects under Grant number ZDKJ202004 and the Department of Science and Technology of Hainan Province, and Key Research and Development Program under Grant number ZDYF2020112. The funder had no role in the study design or implementation, data collection, management, analysis, interpretation, manuscript preparation, review, or approval, or the decision to submit the manuscript for publication.

## Conflict of Interest

The authors declare that the research was conducted in the absence of any commercial or financial relationships that could be construed as a potential conflict of interest.

## Publisher's Note

All claims expressed in this article are solely those of the authors and do not necessarily represent those of their affiliated organizations, or those of the publisher, the editors and the reviewers. Any product that may be evaluated in this article, or claim that may be made by its manufacturer, is not guaranteed or endorsed by the publisher.

## Abbreviations

ED: emergency department
ERI: effort-reward imbalance
ERR: effort-reward ratio
OR: odds ratios
95% CI: 95% confidence intervals.
