# Supplementary material for: Effort-Reward Imbalance in Emergency Department Physicians: Prevalence and Associated Factors
Source: Front Public Health. 2022 Feb 7;10:793619. doi: 10.3389/fpubh.2022.793619 (PMC8858846; doi:10.3389/fpubh.2022.793619)
Supplement: Supplementary file 1 [file Table_1.DOCX]

**Table 1:** Characteristics of included studies

| Study | Year | No. of HF patients | No. of controls | HR | Adjusted factors |
| --- | --- | --- | --- | --- | --- |
| Kwak^a^ | 2021 | 101924 | 578266 | 1.09 (1.05-1.13) | age, sex, income, diabetes mellitus, smoking, alcohol consumption, and body mass index. |
| Leedy | 2021 | 3272 (female only) | 146817 | 1.28 (1.11-1.48) | BMI, diabetes, smoking, age at enrollment, baseline PCP visit within 1 year, physical activity, alcohol, ethnicity, education, income, hormone use ever, hypertension, cardiac medication use, family history of cancer, history of CVD and high cholesterol. |
| Roderburg | 2021 | 100124 | 100124 | 1.76 (1.71-1.81) | sex, age, index year, obesity, diabetes and consultation frequency. |
| Schwartz | 2020 | 167633 | 837126 | 0.93 (0.91–0.96) | age, sex, baseline prevalence of ischemic heart disease (including prior MI), diabetes, COPD, liver disease, and chronic kidney disease as present at baseline, baseline use of aldosterone antagonists, beta blockers, ACE-inhibitors, oral anticoagulation and antiplatelet therapy. |
| Selvaraj | 2018 | 1420 (male only) | 26921 | 1.05 (0.86-1.29) | race, cigarette smoking, alcohol use, aspirin use, family history of cancer, cirrhosis, proton pump inhibitor or H_2_ blocker use, sun exposure, any colonoscopy or sigmoidoscopy, physical exam, rectal exam, and prostate specific antigen level tested. |
| Banke^b^ | 2016 | 9307 | NA | 1.14 (1.05-1.24) | gender, age, and date. |
| Hasin | 2016 | 228 | 853 | 1.71 (1.07-2.73) | age, sex, and Charlson comorbidity index. |
| Hasin | 2013 | 596 | 596 | 1.68 (1.13-2.50) | BMI, smoking, and the Charlson comorbidity index. |

a: 2-year lag analysis

b: exclusion of all diagnoses of cancer within the first 365 days
